# Coexistence of nonfluorescent chromoproteins and fluorescent proteins in massive *Porites* spp. corals manifesting a pink pigmentation response

**DOI:** 10.3389/fphys.2024.1339907

**Published:** 2024-06-17

**Authors:** Toshiyuki Suzuki, Beatriz E. Casareto, Mathinee Yucharoen, Hideo Dohra, Yoshimi Suzuki

**Affiliations:** ^1^ Graduate School of Science and Technology, Shizuoka University, Shizuoka City, Japan; ^2^ Faculty of Environmental Management, and Coastal Oceanography and Climate Change Research Center, Prince of Songkla University, Songkhla, Thailand; ^3^ Research Institute of Green Science and Technology, Shizuoka University, Shizuoka City, Japan

**Keywords:** chromoprotein, fluorescent protein, non-fluorescent chromoprotein, *Porites* spp., oxidative stress, pink pigmentation response, innate immune response

## Abstract

**Introduction:**

Several fluorescent proteins (FPs) and chromoproteins (CPs) are present in anthozoans and play possible roles in photoprotection. Coral tissues in massive corals often display discoloration accompanied by inflammation. Incidences of the pink pigmentation response (PPR) in massive *Porites*, described as inflammatory pink lesions of different shapes and sizes, has recently increased worldwide. FPs are reported to be present in PPR lesions, wherein a red fluorescent protein (RFP) appears to play a role in reducing reactive oxygen species. However, to date, the biochemical characterization and possible roles of the pigments involved are poorly understood. The present study aimed to identify and characterize the proteins responsible for pink discoloration in massive *Porites* colonies displaying PPRs, as well as to assess the differential distribution of pigments and the antioxidant properties of pigmented areas.

**Method:**

CPs were extracted from PPR lesions using gel-filtration chromatography and identified via genetic analysis using liquid chromatography-tandem mass spectrometry. The coexistence of CPs and RFP in coral tissues was assessed using microscopic observation. Photosynthetic antivity and hydrogen peroxide-scavenging activitiy were measured to assess coral stress conditions.

**Results:**

The present study revealed that the same CP (plut2.m8.16902.m1) isolated from massive *Porites* was present in both the pink spot and patch morphologies of the PPR. CPs were also found to coexist with RFP in coral tissues that manifested a PPR, with a differential distribution (coenosarc or tip of polyps’ tentacles). High hydrogen peroxide-scavenging rates were found in tissues affected by PPR.

**Discussion and Conclusion:**

The coexistence of CPs and RFP suggests their possible differential role in coral immunity. CPs, which are specifically expressed in PPR lesions, may serve as an antioxidant in the affected coral tissue. Overall, this study provides new knowledge to our understanding of the role of CPs in coral immunity.

## 1 Introduction

Global climate change and anthropogenic disturbances have led to an increased incidence of coral diseases and bleaching during the last 3 decades, particularly in corals inhabiting tropical regions in the Caribbean and Indo-Pacific Ocean (Beeden et al., 2008; Aeby et al., 2011; Weil et al., 2012; 2016; Hughes et al., 2018; Johnson et al., 2022). In the last decade, heat-susceptible genotypes may have declined and/or adapted to such conditions; therefore, the remaining coral populations could have a higher thermal threshold for bleaching (Sully et al., 2019). Despite the increasing risk of climate change affecting coral health, studies have revealed that corals can improve their resilience through certain innate physiological mechanisms that involve chaperones and antioxidative enzymes, particularly heat shock proteins (Hsp70, Hsp60, and Hsp32) (Arya et al., 2007; Lanneau et al., 2008; Jin et al., 2016; Seveso et al., 2018; 2020; Montalbetti et al., 2021; Thummasan et al., 2021).

Massive *Porites* spp. are reef-building corals commonly found in reefs of the Indo-Pacific region, including the Ryukyu Islands in southern Japan (Veron, 1992; Nishihira, 2004). The health compromised coral tissues in massive corals such as *Porites* spp. often display non-normal pigmentation and develop some discoloration, such as pink, purple, and blue, that potentially represent an inflammation-like response (Palmer et al., 2009a). Among these, the pink pigmentation response (PPR) in massive *Porites* spp. colonies, a generalized inflammatory response characterized by the formation of pink lesions of different shapes and sizes, has been reported to increase in the Ryukyu Islands (Kubomura et al., 2021), with chronic seasonal variations (Yucharoen, 2016). In general, PPRs in corals develop swollen areas with pink discoloration, described as “pink lines” (Ravindran and Raghukumar 2002; 2006; Kenkel, 2008; Mohamed et al., 2012; Kritsanapuntu and Angkhananukroh, 2014; Séré et al., 2015), “pink spots” (Willis et al., 2004; Benzoni et al., 2010; Thinesh et al., 2011; Putchim et al., 2012; Kumar et al., 2014), and “pink pigmentation” (Raymundo et al., 2005; Palmer et al., 2009a).

Discoloration in PPRs is a phenomenon frequently observed in *Porites* spp. corals, but there are some reports regarding similar pigmentation phenomenon in other genera, such as *Acropora* (Bongiorni and Rinkevich, 2005; D’Angelo et al., 2012) and *Montipora* (D’Angelo et al., 2012; Aeby 1992; 2003 found that corals manifesting pink spots were infected by the trematode *Podocotyloides stenometra*. However later in the literature; Benzoni et al., 2010; Yucharoen 2016, found that the pink spots and other pink swollen lesions observed in massive corals, may not always be the result of tissue inflammation caused by the trematodes, but also injuries caused by other organisms penetrating the coral tissues, bites of fishes and the response to environmental stressors like high sea-surface temperature, strong illumination, low salinity, and desiccation. Therefore, the PPR is linked with an immune response of corals to those stresses mainly promoting the formation of reactive oxygen species (ROS) and the coral innate immune response through the production of antioxidant enzymes and FP (fluorescent proteins) (Palmer et al., 2009a; Palmer et al., 2009b; Bridges et al., 2020). Palmer et al. (2009)a and Palmer et al. (2009)b reported the presence of a red fluorescent protein (RFP) that plays an important role in photoprotection and the reduction of ROS. Bridges et al. (2020) reported that RFP expression in pink-pigmented lesions of *Porites lobata* is an innate immune response. Moreover, green fluorescent proteins (GFPs) in scleractinian corals play an important role in the removal of ROS (Bou-Abdallah et al., 2006).

To date, several FPs and chromoproteins (CPs) have been reported in anthozoans with possible roles in photoprotection (Matz et al., 1999; Miyawaki, 2002; Verkhusha and Lukyanov 2004; Bollati et al., 2022). The CPs present in anthozoans are highly homologous to FPs, such as GFPs. FPs and FP-like CPs are classified into four groups: cyan, green, and red that are fluorescent, and nonfluorescent CPs (Labas et al., 2002). Apart from the normal green-brown color of anthozoans from their symbiotic algae, CPs are also responsible for the nonfluorescent coloration (Lukyanov et al., 2000; Wiedenmann et al., 2000). Since the discovery of GFPs in the crystal jelly, *Aequorea victoria*, CPs and FPs have been extensively studied. Alieva et al. (2008) revealed for the first time that several CPs are present in some scleractinian coral genomes. Similarly, Shinzato et al. (2012) reported the presence of fluorescent and nonfluorescent CPs in a whole-genome analysis of *Acropora* corals. In recent years, it has become clear that diverse FPs are present with complex expression in corals of the genus *Acropora* (Kashimoto et al., 2021; Satoh et al., 2021). However, it is not clear what condition will promote the expression of these proteins in *Porites* spp. corals. Therefore, this study aimed to identify and characterize the CPs expressed in the discolored tissues of massive *Porites* spp. colonies manifesting PPRs, by extracting and purifying their proteins and further identifying the genes involved in their expression. In addition, microscopic observations of coral tissues under normal and ultraviolet light were conducted to clarify differences in the localization of CPs and RFP.

## 2 Materials and methods

### 2.1 Coral sampling

**FIGURE 1:**
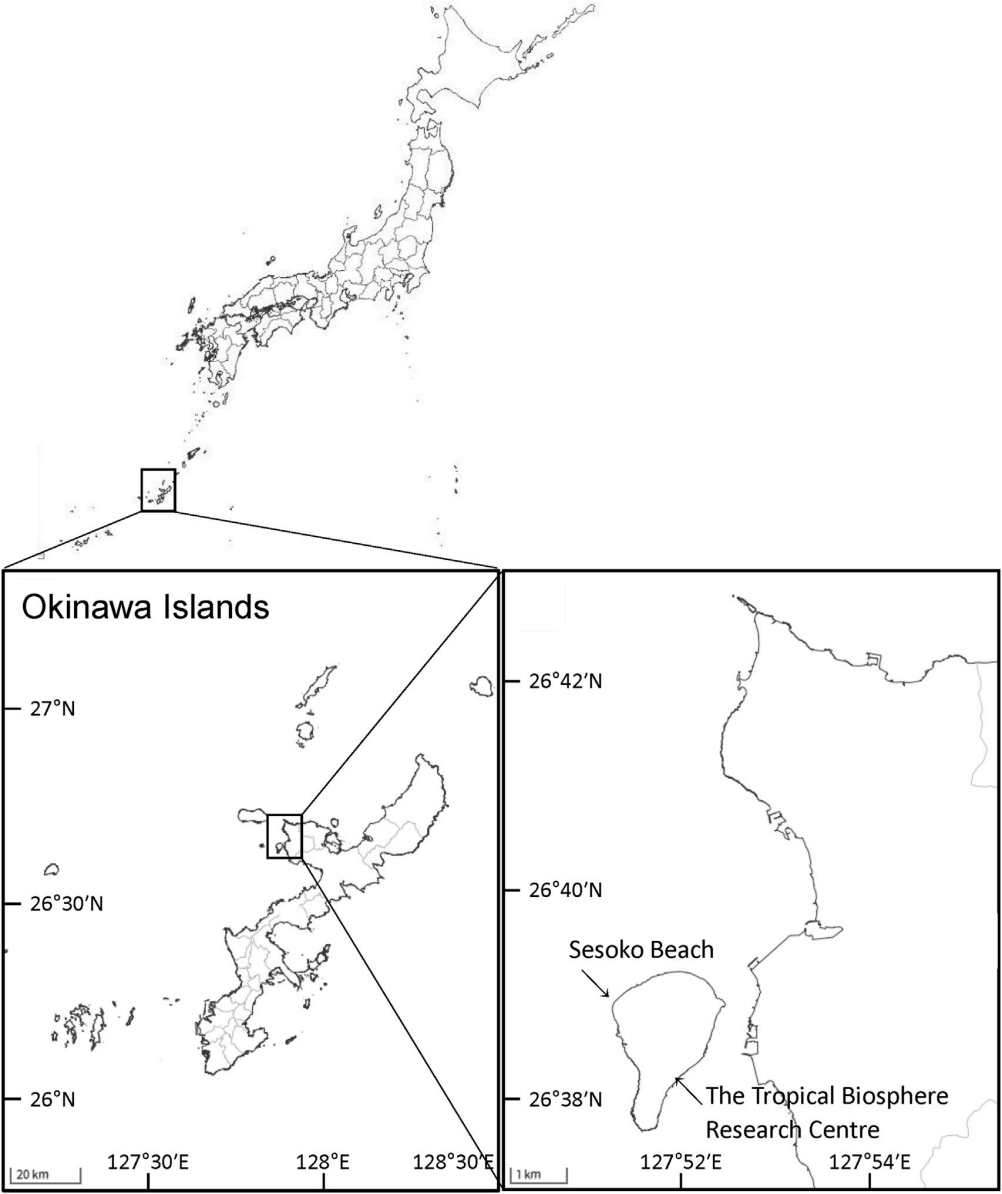
Study area and sampling sites in Okinawa, Japan. Maps were obtained from The Geospatial Information Authority of Japan (https://www.gsi.go.jp/).

**FIGURE 2:**
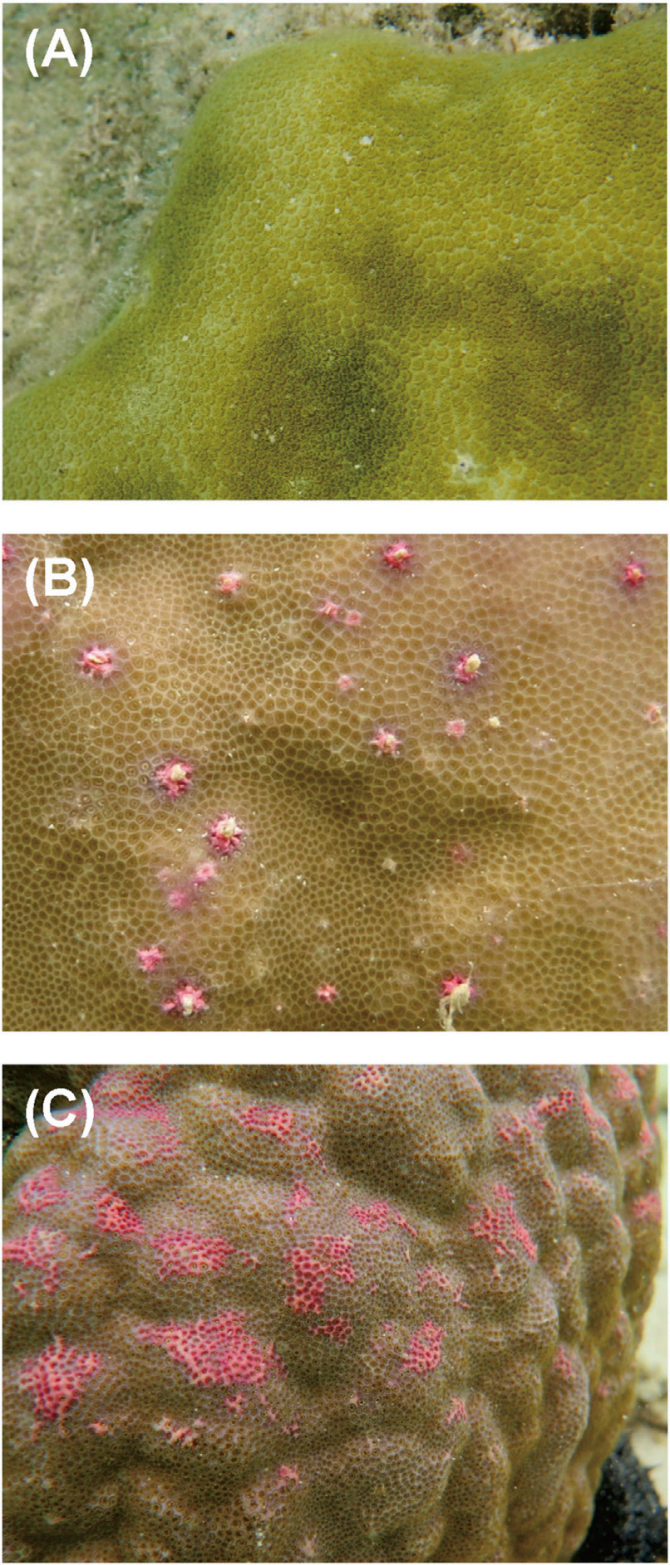
Aspects of different types of PPRs in massive *Porites* colonies: **(A)** healthy-appearing colony, **(B)** colony exhibiting pink spots, and **(C)** colony exhibiting pink patches.

Colonies of massive *Porites* spp. were sampled from a coral reef at Sesoko Beach in Sesoko Island, Okinawa, Japan (26°39ʹN 127°51ʹE), at low tide (depth 0.5–1 m) on 18 September 2018 (sampling permit No. 30–55, granted by the Okinawa Prefectural Government) (Figure 1). For protein analysis, a hammer and chisel were used to collect coral pieces from three colonies of *Porites* spp., two displaying PPRs as pink spots (Ps) and pink patches (Pp), and one healthy colony (H) (Figure 2). For microscopic observation, the coral pieces displaying Pp and the healthy-appearing colony were used. Sampled fragments of each colony (10–20 cm^2^) were kept in sterilized tagged bags with surrounding seawater and transported to the laboratory at the Tropical Biosphere Research Centre (University of The Ryukyus, Okinawa, Japan) at Sesoko Island. Samples were maintained in an aquarium for 24 h with natural running seawater, temperature fluctuating between 26.5°C and 27.5°C, and attenuated natural illumination with a maximum of 200 μmol photons cm^−2^ s^−1^ to avoid the formation of ROS until treatment.

### 2.2 Extraction and concentration of chromoproteins

**FIGURE 3:**
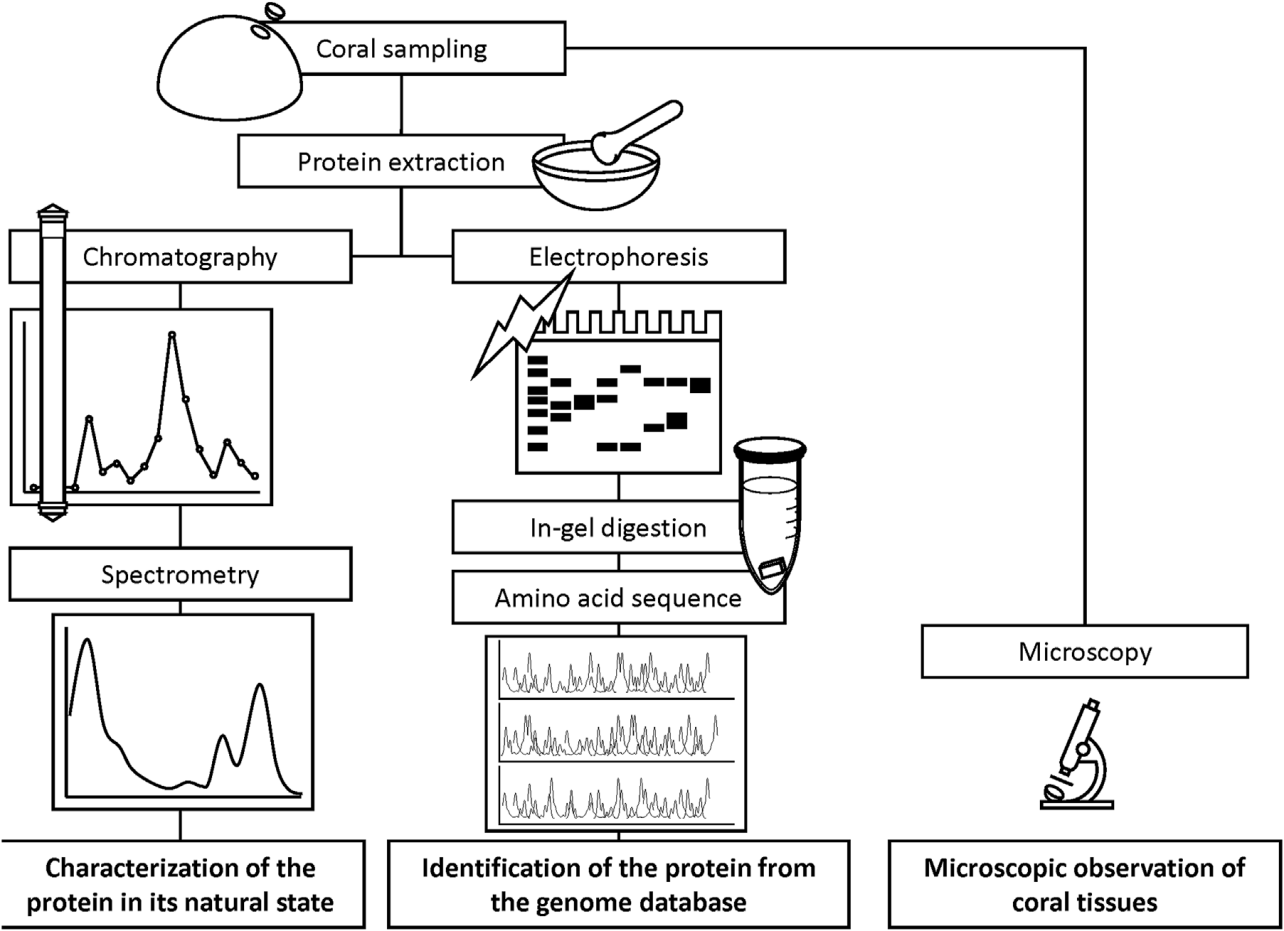
Schematized experimental flow for the characterization of chromoprotein.

Tissue solutions were obtained from the pink-pigmented area of the colonies displaying Ps and Pp, as well as from H as a control. Coral tissues of 0.8–1.6 cm^2^ (Ps), 3–5 cm^2^ (Pp), and 4 cm^2^ of H were removed from the skeleton with a Waterpik dental flosser (DENTREX; Ricoh Elemex, Aichi, Japan) (Johannes and Wiebe, 1970) using 3.5% NaCl solution and 10 mM sodium phosphate buffer (pH 7.5). Crude tissue solutions were homogenized using a glass homogenizer and filtered through Whatman glass microfiber filters (Grade GF/F, pore size 0.7 µm; GE Healthcare Life Sciences, Buckinghamshire, United Kingdom) to remove the endosymbiotic algae Symbiodiniaceae cells and coral tissue debris. The soluble fractions were centrifuged at 3,000 × *g* using Amicon Ultra centrifugal filters (Merck KGaA, Darmstadt, Germany) to concentrate molecules with masses greater than 10,000 Da to retain proteins. Concentrated extracts were transferred to microtubes using Pasteur pipettes. The Amicon meshes were then washed three times with 200 µL of 10 mM sodium phosphate buffer (pH 7.5) for complete recovery of the samples. The final volume of the extract was approximately 1.5 mL. Approximately 50 μL of the protein extracts were used for separation through electrophoresis, and the remaining used for purification. A scheme of the experimental flow is shown in Figure 3.

### 2.3 Purification and characterization of chromoproteins

Partial purification of CPs from the protein extracts was performed via successive gel-filtration chromatography (Superdex 200 HR 10/30 column; GE Healthcare Life Science) on a fast protein liquid chromatography (FPLC) system (ÄKTA prime; GE Healthcare Life Science). The samples were eluted with 10 mM sodium phosphate buffer (pH 7.5) containing 0.15 M NaCl at a flow rate of 0.5 mL/min. Forty elution products were collected in 1-mL fractions each, and their optical properties and enzymatic activity measured. Absorbance at 280 and 580 nm was measured for each fraction using a spectrophotometer (model UV-2450; Shimadzu, Kyoto, Japan). Molecular weights were estimated using a Gel Filtration Standard molecular marker (Bio-Rad Laboratories, Hercules, California, United States of America).

### 2.4 Amino acid sequence identification

#### 2.4.1 Protein concentration

The total protein content was determined using a Bio-Rad Protein Assay Kit (Bio-Rad Laboratories) based on the Bradford method (Bradford, 1976) with bovine serum albumin as a standard. The Bradford method quantifies proteins using Coomassie Brilliant Blue G-250, a triphenylmethane blue dye that binds to proteins in acidic solutions and shifts the maximum absorption wavelength from 465 to 595 nm. Absorbance at 595 nm was measured using a Shimadzu spectrophotometer (model UV-2450) at 25°C.

#### 2.4.2 Electrophoresis

Sodium dodecyl polyacrylamide gel electrophoresis (SDS-PAGE) was performed using a RAPIDAS AE-6500 apparatus (ATTO, Tokyo, Japan) with 1-mm thick precast slab gels containing 10%–20% gradient acrylamide (e-PAGEL E-T1020L; ATTO), according to the procedure described by Laemmli (1970). 100 μg of each protein extract (Pp, Ps, and H) were loaded onto the gel. Electrophoresis was performed at 40 mA for 80 min. After electrophoresis, the gel was stained with a CBB-250 solution (Wako Pure Chemical Industries, Osaka, Japan). The applied molecular mass standard (Precision Plus Protein Standards; Bio-Rad Laboratories) contained protein bands of 10, 20, 25, 37, 50, 75, 100, 150, and 250 kDa in size. Gel photographs were taken with a Panasonic DMC-GF3 camera (Panasonic, Osaka, Japan), and the molecular weights of the protein sample bands calculated using ImageJ software (http://imagej.net/) after the exposure and contrast were adjusted.

#### 2.4.3 In-gel tryptic digestion

The protein bands obtained from the CBB-stained SDS-PAGE gel were sliced into small pieces (approximately 1 mm^3^) and faded with 50 mM NH_4_HCO_3_/50% acetonitrile. Proteins in the sliced gel fragments were reduced and alkylated with 10 mM dithiothreitol/50 mM NH_4_HCO_3_ for 45 min at 56°C and 55 mM iodoacetamide/50 mM NH_4_HCO_3_ at 25°C for 30 min in the dark. After being washed with acetonitrile, the proteins were digested in 10 µL trypsin (Sequencing Grade; Promega, Madison, WI, United States of America) at 37°C overnight. Tryptic peptides were extracted from the gel fragments using 50% acetonitrile containing 3% formic acid, and the peptide extracts centrifuged at 10,000 × *g* for 20 min in a vacuum centrifuge.

#### 2.4.4 Liquid chromatography-tandem mass spectrometry analysis

Liquid chromatography-tandem mass spectrometry analysis was performed using a linear ion trap time-of-flight mass spectrometer (LIT–TOF MS; NanoFrontier eLD; Hitachi High-Technologies Corporation, Tokyo, Japan) coupled to a nanoflow HPLC (NanoFrontier nLC; Hitachi High-Technologies Corporation) at the Instrumental Research Support Center of the Research Institute of Green Science and Technology, Shizuoka University (Shizuoka, Japan). Peptides extracted from the gel were trapped and desalted using a C18 monolith trap column (0.05 mm ID × 150 mm long; Hitachi High-Technologies Corporation) and thereafter loaded onto a MonoCap C18 Fast-flow column (0.05 mm ID × 150 mm long; GL Sciences, Inc., Tokyo, Japan). Elution was done using a linear gradient from 5% to 40% solvent B for 60 min at a flow rate of 200 nL/min. Solvent A comprised 2% acetonitrile and 0.1% formic acid, and solvent B contained 98% acetonitrile and 0.1% formic acid. The eluent was ionized using a nanoelectrospray ionization source equipped with an uncoated silica tip (New Objective, Woburn, MA, United States of America) and analyzed using LIT–TOF MS. Mass spectra were obtained by scanning in the positive ion mode in the mass range of *m/z* 200–2000. MS/MS spectra were generated by using collision-induced dissociation in a linear ion trap. The proteins were identified by analyzing the MS/MS data via *de novo* sequencing and with the protein identification software, PEAKS Studio (version 7.0; Bioinformatics Solutions, Inc., Waterloo, ON, Canada) (Ma et al., 2003). A protein identification database was generated using the protein sequences of *P. astreoides*, *P. australiensis*, and *P. lobata* obtained from Reef Genomics (http://reefgenomics.org/) (Liew et al., 2016). Reliability of the protein identification results obtained via PEAKS was increased through further analysis of the sequences using MASCOT MS/MS Ions Search (http://www.matrixscience.com/) (Cottrell, 2011) and the sequence tag search tool, SPIDER (Han et al., 2004). The detected proteins were identified through a similarity search of amino acid sequences using the Basic Local Alignment Search Tool (BLAST) on the National Center of Biotechnology Information database (https://blast.ncbi.nlm.nih.gov/), the DNA Data Bank of Japan (DDBJ) (http://blast.ddbj.nig.ac.jp/), and Reef Genomics.

#### 2.4.5 Phylogenetic analysis

A phylogenetic tree of the proteins identified in this study, together with other FPs described previously (Alieva et al., 2008), was constructed using an online program (http://www.phylogeny.fr/) developed by Dereeper et al. (Dereeper et al., 2008; Dereeper et al., 2010). Phylogenetic analysis was performed using the following programs: MUSCLE 3.8.31 for multiple alignments, Gblocks 0.91b for alignment refinement, PhyML 3.1 for phylogeny using maximum likelihood, and TreeDyn for tree rendering.

### 2.5 Microscopic observation of *in vivo* fluorescence in corals

Overview photographs of corals manifesting PPR and healthy-appearing corals were taken in the laboratory with an OLYMPUS E-PL6 camera under a white light-emitting diode (LED) and processed using Adobe Lightroom (Adobe, San Jose, CA, United States of America) with the following settings: exposure, +0.5; highlight, −75; shadow, −20; white level, +65; black level, −40. The fluorescence levels of each sample were observed using a stereoscopic microscope (Nikon SMZ-1000; Nikon, Tokyo, Japan), and photographs obtained using an OLYMPUS E-PL6 camera equipped with a mount conversion ring. Visible images were obtained under environmental light and an exposure of one-fifth of a second with a sensitivity of ISO 400. Fluorescence images were obtained through excitation with a blue LED with a wavelength range of 380–550 nm (KR93SP; Eco-lamps Inc., Kowloon, Hong Kong), exposure for 13 s with a sensitivity of ISO 100, and filtering through a 600-nm bandpass filter (BPB-60; Fujifilm, Tokyo, Japan). The bandpass filter was attached to the objective lens of the microscope. All images were processed using Adobe Lightroom software.

### 2.6 Maximum quantum yield of photosystem II

The photosynthetic activity of Symbiodiniaceae under each coral condition was evaluated using the maximum quantum yield of photosystem II (*F*v/*F*m), and the minimum fluorescence yield (*F*0) measured using Junior-PAM (Walz, Effeltrich, Germany). Coral nubbins were subjected to dark acclimation for 30 min (Krause and Weis, 1991). The *F*v/*F*m and *F*0 values measured at 10 different points in every coral nubbin were used to estimate averages and standard errors (Supplementary Table S1).

### 2.7 Hydrogen peroxide-scavenging activity

Enzyme samples were prepared by removing excess water from the collected coral pieces and excavating nine points (1.0–1.5 cm^2^) on each coral colony surface (Pp and H) using whetstone drilling. The excavated areas were rinsed with 1 mL of 50 mM Tris-HCl buffer (pH 7.6) to collect the coral tissue, and the extract centrifuged at 10,000 × *g* for 3 min to remove debris. The supernatant was used to measure the protein concentration and hydrogen peroxide-scavenging activity (HSA), following the method described in Palmer et al., 2009a. To measure HSA, each 50 µL of the sample (coral tissue in buffer solution) and 50 µL of 50 mM hydrogen peroxide solution were mixed on 96-wells microplate. The absorbance at 240 nm was measured every 81 s for 30 min, and the data converted using a standard curve of hydrogen peroxide (serial dilutions: 0, 6.25, 12.5, 25, and 50 mM). HSA was normalized to the protein concentration (Supplementary Table S2).

### 2.8 Statistical analysis

Independent-samples *t*-test analysis was carried out to determine significant differences between the *F*v/*F*m and HSA data from Pp colony and H colony. Normality of the data was tested using Anderson–Darling test. Significant differences were evaluated at *p* < 0.01.

## 3 Results

### 3.1 Purification and characterization of chromoproteins

**FIGURE 4:**
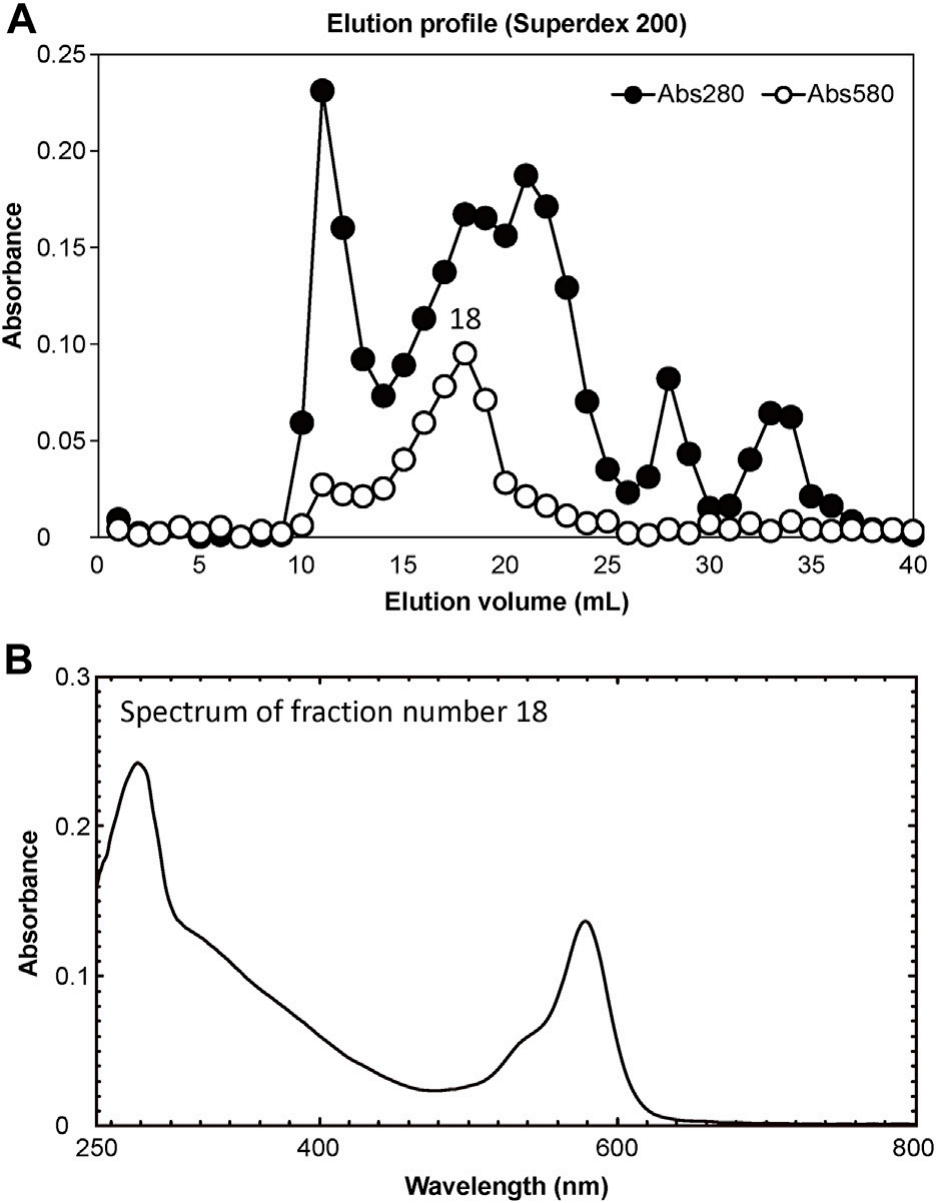
Results of purification of extracted proteins. **(A)** Separation of pink-coloured protein by Superdex 200 gel filtration column chromatography. Solid and open circles indicate protein fractions measured at 280 nm (estimate protein content) and 580 nm (estimate CP content), respectively. **(B)** Absorption spectrum of peak fraction number 18 in the elution profile.

The soluble fraction of coral tissues manifesting Pp was separated into 40 elution fractions using FPLC. Of these, elution product 18 (18 mL elution volume) showed the highest absorbance at a wavelength of 580 nm (Figure 4A). Using a marker protein, the molecular weight of the main peak fraction (18) was estimated to be 49 kDa. The absorption spectrum of the main fraction is shown in Figure 4B.

### 3.2 Identification of chromoproteins from corals manifesting pink pigmentation responses

**FIGURE 5:**
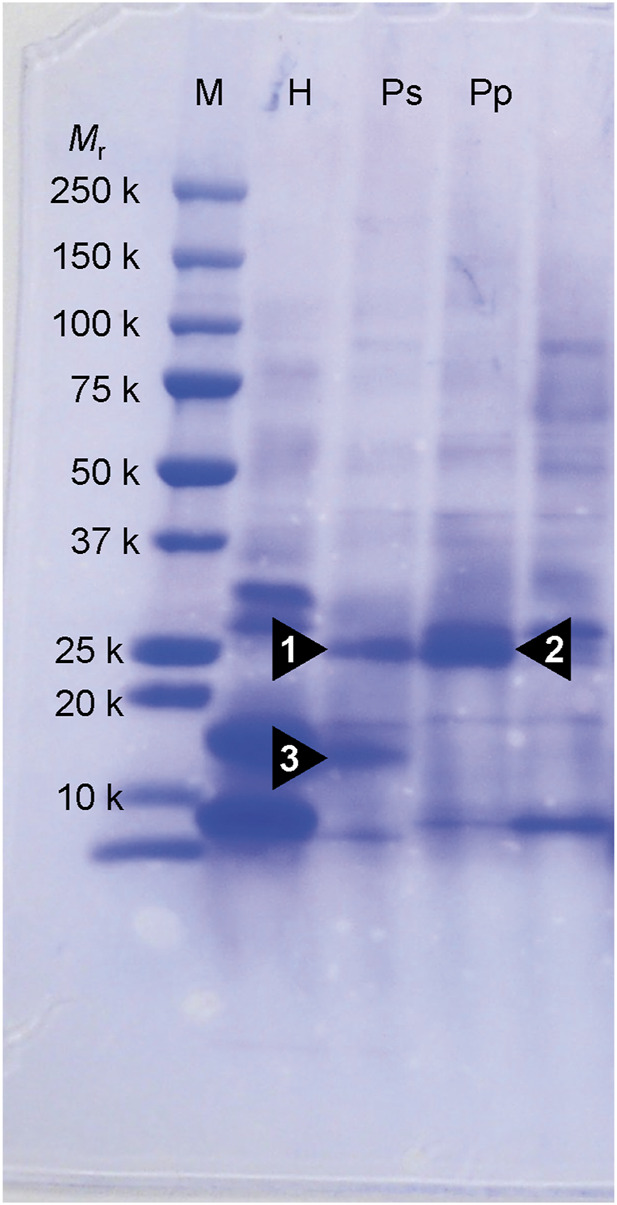
SDS-PAGE on 10% polyacrylamide gel of partially purified protein extracts from corals. Proteins were stained by coomassie brilliant blue R-250. Numerals under *M*r denote the molecular masses of marker protein. Arrowhead (1, 2 and 3) indicates a protein that appears specifically in the pink lesions of Ps and Pp. M, molecular weight markers; H, healthy-appearing colony; Ps, pink spot exhibiting colony; Pp, pink patch exhibiting colony.

**FIGURE 6:**
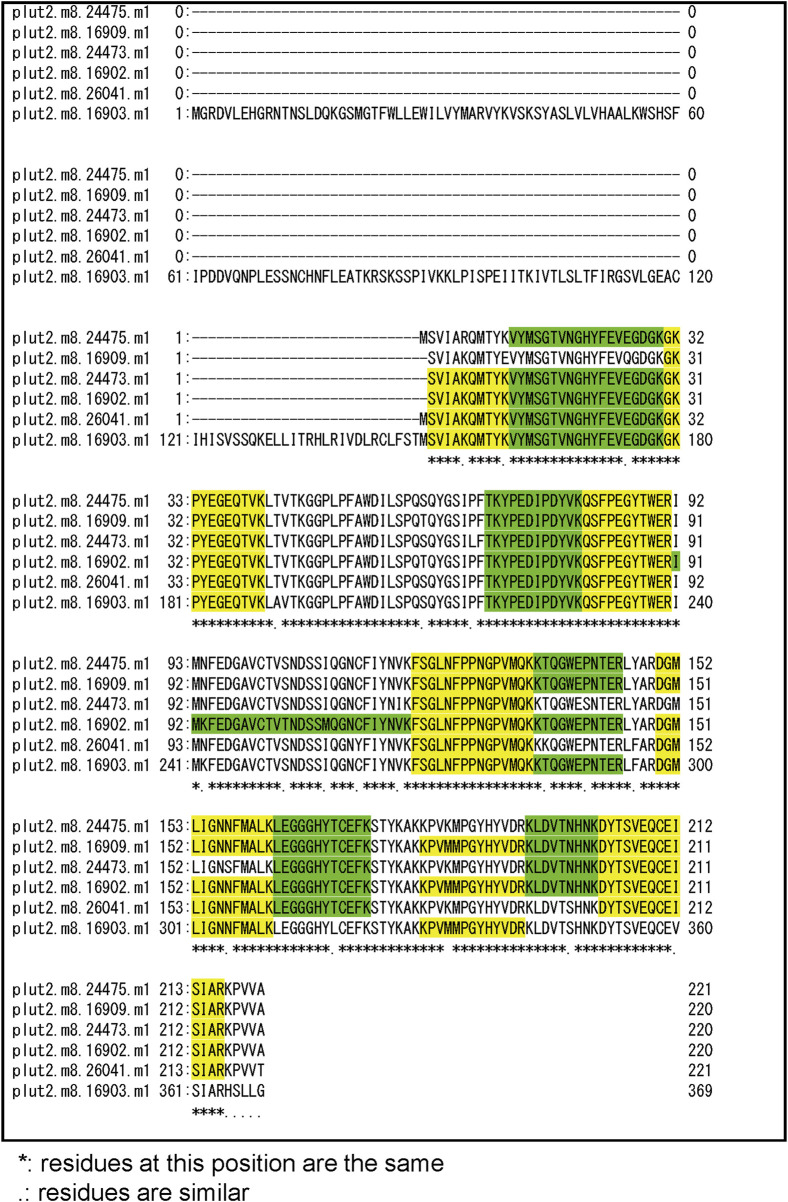
Alignment of amino acid sequence of 6 candidate proteins that hitted with high coverage values for peptide fragments of bands 1 and 2 (Protein A) in Figure 5. Peptide fragments detected by PEAKS Software were indicated as green and yellow.

**FIGURE 7:**
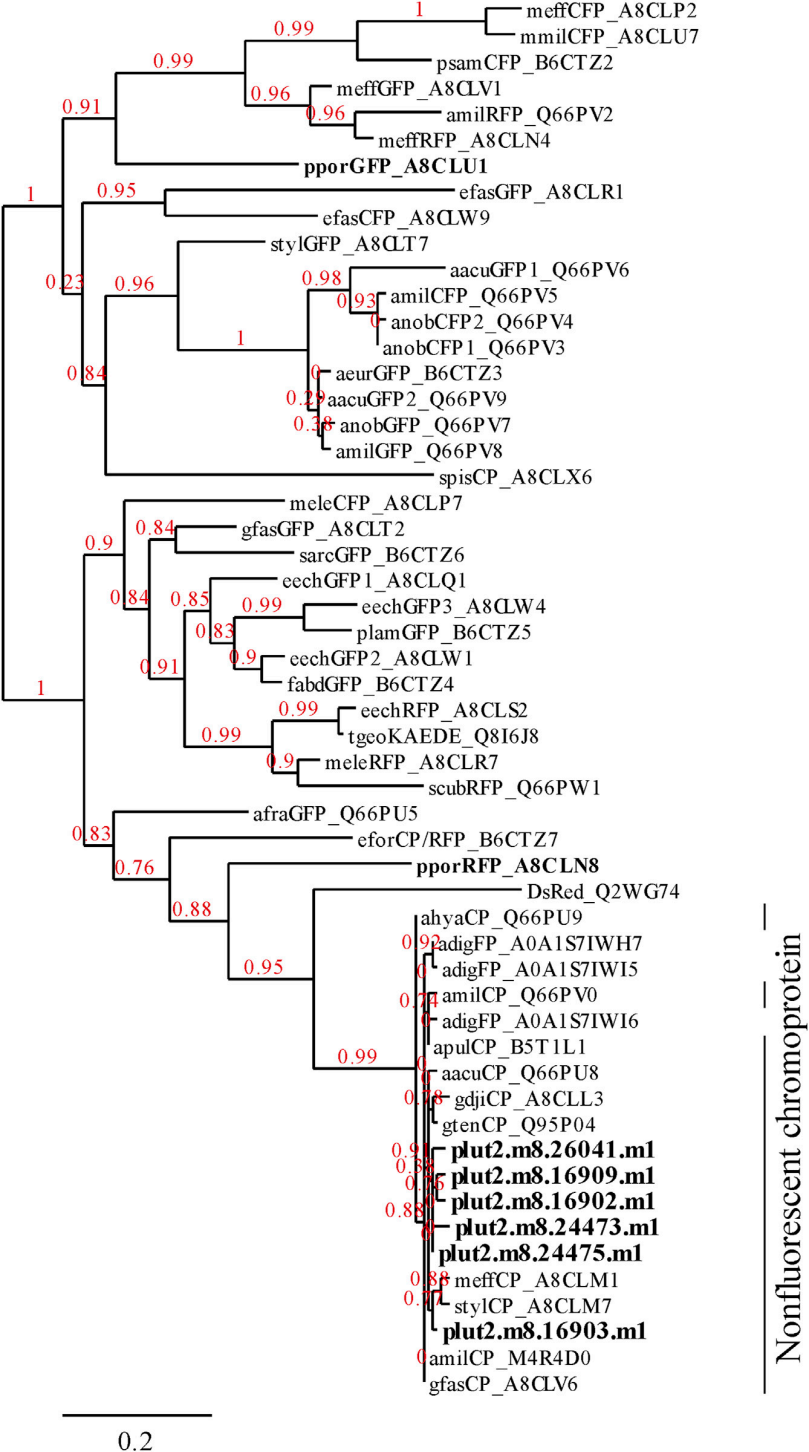
Phylogenetic tree showing the relationships among 6 proteins found in the present research (Figure 6) and FPs and CPs of corals. The alignments of *P. lutea* protein were identified using PEAKS analysis. Other FPs listed in Alieva et al. (Alieva et al., 2008) were obtained from the database of UniProt (http://www.uniprot.org/). The 6 proteins identified in this study and other FPs of *Porites* spp. are indicated in bold text.

Three notable bands on the SDS-PAGE gel were found in the Ps and Pp coral tissues (Figure 5; bands 1–3). PEAKS analysis showed that the proteins extracted from bands 1 and 2 (Protein A) had the same alignment as that of the candidate protein (Porites_australiensis_11250) that was detected in the protein sequences of *P. australiensis* with high coverage (75%). The amino acid sequence of Protein A was queried on Reef Genomics with the protein database “Porites lutea gene models (ReFuGe 2020),” which identified 20 homologous proteins. Reanalysis of the Protein A was performed with these 20 proteins using PEAKS, where the database identified six proteins with high scores and coverage values with respect to the proteins synthesized by *P. lutea*. The amino acid sequences of these six proteins are shown in Figure 6. Among the six proteins, plut2.m8.16902.m1 with a GFP domain had the highest coverage (81%). Alignment of plut2.m8.16902.m1 using the BLAST search program in the DDBJ database revealed high similarities with other CPs and FPs from *Acropora aculeus*, *A. digitifera*, *Goniopora tenuidens*, *Galaxea fascicularis*, *A. millepora*, and many other coral species (Table 1). Using electrophoresis and amino acid sequence analyses (in-gel trypsin digestion and liquid chromatography-tandem mass spectrometry analysis), its molecular weight was determined to be 25 kDa (Figure 5; Table 1). The protein in band 3 (Protein B) was also analyzed using the same methods and showed a high similarity with thioredoxin-like proteins from several marine organisms (Table 1). On the other hand, no remarkable band in the electrophoresis result near 25 kDa (CPs and GFP) was found for the healthy colony: the greenish appearance of the healthy colony (Figure 2A) was due to the reflection from the green band of the endolithic community that was well developed underneath the coral tissue and not to the presence of GFP. Moreover, the molecular weight of GFP in *Porites lutea* is 24.8 kDa (*Porites lutea* database in Reef Genomics) but, from the electrophoresis study, the healthy coral did not show high expression of protein in the band corresponding to GFP’s molecular weight. Phylogenetic analysis revealed that the six identified proteins were nonfluorescent, similar to that of other CPs in several coral species (Figure 7).

**TABLE 1:** Amino acid sequences and homologues of protein that were specific for corals manifesting Ps and Pp. Alignments were determined by BLAST search from database of *P. lutea* on Reef Genomics. Putative conserved domains and similar proteins were determined by BLAST search from UniProtKB/Swiss-Prot + TrEMBL database on DDBJ.

[Bands 1 and 2; protein A]				[Band 3; Protein B]			
Alignment				Alignment			
Name: plut2.m8.16902.m1 Alignment: SVIAKQMTYKVYMSGTVNGHYFEVEGDGKGKPYEGEQTVKLTVTKGGPLPFAWDILSPQTQYGSIPFTKYPEDIPDYVKQSFPEGYTWERIMKFEDGAVCTVTNDSSMQGNCFIYNVKFSGLNFPPNGPVMQKKTQGWEPNTERLYARDGMLIGNNFMALKLEGGGHYTCEFKSTYKAKKPVMMPGYHYVDRKLDVTNHNKDYTSVEQCEISIARKPVVA				Name: plut2.m8.16752.m1 Alignment: QDDFDAFLKEAGSTLVVVDFYADWCGPCKMIAPKIKGFAEEFSGKVYFAKVNVDENDEVAGKEGISAMPTFNLYKNGAKVDELTGANEAKLRELIEKRI			
Average mass: 24,870				Average mass: 10,957			
Putative conserved domains: GFP superfamily				Putative conserved domains: TRX_family, thioredoxin and thioredoxin_like superfamily			
Similar proteins				Similar proteins			
Accession number	Protein name	Species	Identity	Accession number	Protein name	Species	Identity
Q66PU8	Chromoprotein	*Acropora aculeus*	211/220 (95%)	A0A2B4SRX0	Thioredoxin	*Stylophora pistillata*	61/94 (64%)
A0A1S7IWH7	Fluorescent protein	*Acropora digitifera*	211/220 (95%)	A0A1X7VQB2	Thioredoxin	*Amphimedon queenslandica* (sponge)	56/97 (57%)
Q95P04	GFP-like non-fluorescent chromoprotein	*Goniopora tenuidens*	211/220 (95%)	A0A3S3QB26	Thioredoxin	*Dinothrombium tinctorium* (red velvet mite)	58/98 (59%)
A8CLV6	GFP-like chromoprotein	*Galaxea fascicularis*	211/220 (95%)	A0A1D2NA26	Thioredoxin-2	*Orchesella cincta* (springtail)	61/98 (62%)
A0A1S7IWI5	Fluorescent protein	*Acropora digitifera*	210/220 (95%)	L7XAL0	Thioredoxin	*Scylla paramamosain* (mud crab)	58/97 (59%)
Q66PV0	Chromoprotein	*Acropora millepora*	210/220 (95%)	Accession number	Protein name	Species	Identity
B5T1L1	Chromoprotein CP584	*Acropora pulchra*	210/220 (95%)	A0A2B4SRX0	Thioredoxin	*Stylophora pistillata*	61/94 (64%)
M4R4D0	Chromoprotein 580	*Acropora millepora*	211/220 (95%)	A0A1X7VQB2	Thioredoxin	*Amphimedon queenslandica* (sponge)	56/97 (57%)
A0A1S7IWI6	Fluorescent protein	*Acropora digitifera*	209/220 (95%)	A0A3S3QB26	Thioredoxin	*Dinothrombium tinctorium* (red velvet mite)	58/98 (59%)
A8CLL3	GFP-like chromoprotein	*Goniopora djiboutiensis*	209/220 (95%)	A0A1D2NA26	Thioredoxin-2	*Orchesella cincta* (springtail)	61/98 (62%)

### 3.3 Localization of chromoproteins and red fluorescent protein in corals displaying pink pigmentation responses

**FIGURE 8:**
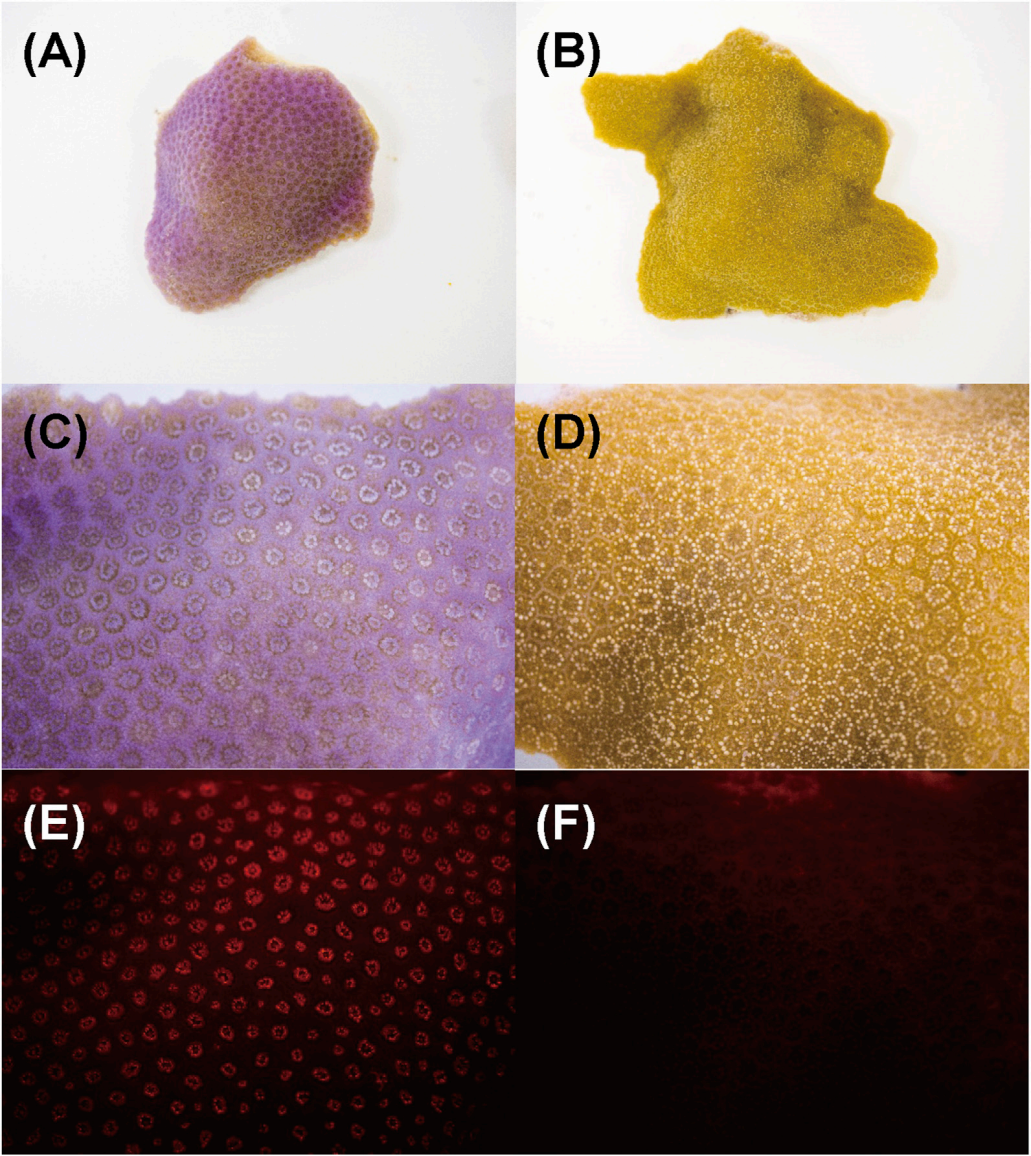
Aspects of *Porites* nubbins and fluorescence image that were taken under microscope. Images of **(A, B)** are overview of corals nubbins and **(C–F)** are magnified images (10X). **(A, C, and E)** Coral manifesting pink patch (Pp), **(B, D and F)** healthy-appearing colony **(H)**. The **(E and F)** are fluorescent image under blue LED light.

Figure 8 shows the aspect of the colony manifesting Pp (Figure 8A,C,E) and healthy-appearing coral colony (Figure 8B,D,F) under visible light, and their fluorescence under a stereoscopic microscope using the bandpass filter transmitting light at 600 nm wavelength. Under visible light, the entire surface of the corals was purple and the tentacles a pale pink (Figure 8C). Conversely, under blue LED light and bandpass filter, red fluorescence was observed only at the tip of the tentacles (Figure 8E), and slight fluorescence was observed in healthy-appearing corals (Figure 8F).

### 3.4 Evaluation of the coral stress state

The stress conditions of the studied corals and their Symbiodiniaceae were estimated by measuring the *F*v/*F*m and *F*0 (Table 2), as well as the HSA (Table 3). The *F*v/*F*m and *F*0 values of the corals with pink pigmentation were 0.434 and 369.5, respectively, which were significantly lower than those of healthy-appearing corals (0.521 and 597.3, respectively) (*p* < 0.01). Furthermore, the HSA value of corals manifesting pink pigmentation (1.88 nmol min^−1^ mg^−1^ protein) was 4.3 times significantly higher than that of healthy-appearing corals (0.44 nmol min^−1^ mg^−1^ protein) (*p* < 0.01).

**TABLE 2:** Comparison of maximum quantum yield of photosystem II (*F*v/*F*m) and minimum fluorescence yield (*F*0) of each coral colony (Pp and H). Means ± s.e. (*n* = 10). Asterisks mean significant differences with healthy-appearing coral by *t*-test at *p* < 0.01.

	*F*v/*F*m	s.e.	*F*0	s.e.
Pp colony	0.434*	±0.036	369.5*	±44.8
H colony	0.521	±0.016	597.3	±25.4

**TABLE 3:** Comparison of hydrogen peroxide scavenging activity (HSA) of each coral colony (Pp and H). Means ± s.d. (*n* = 9). Asterisks mean significant differences with healthy-appearing coral by *t*-test at *p* < 0.01.

	HSA (decrease of nmol H_2_O_2_ min^-1^ mg protein^−1^)	
Pp colony	1.88*	±0.05
H colony	0.44	±0.06

## 4 Discussion

The pink pigment produced by corals manifesting PPRs was found to be a type of nonfluorescent CP. Our results also revealed that the CP was common in Ps and Pp lesions and homologous to other nonfluorescent CPs that have been reported in other corals (Alieva et al., 2008; Shinzato et al., 2012; Takahashi-Kariyazono et al., 2018). Furthermore, Protein A in this study showed high similarity to the six nonfluorescent CPs that are encoded in the genome of *P. lutea*, especially plut2.m8.16902.m1 (plutCP) (Figure 6). In particular, the fragments from I91–K118 were not detected in proteins other than plutCP. In addition, proteins other than nonfluorescent CPs were detected with low coverage and intensity using PEAKS analysis. These results indicate that plutCP expressed in the PPR lesions was a major protein present in the bands 1 and 2. As only plutCP was detected with the highest degree of similarity, we assumed that only this protein could be responsible for the pink-colored tissue. A BLASTP search of the plutCP protein identified several CPs and FPs with high similarities, including CPs of *A. aculeus* (accession number Q66PU8), *A. millepora* (Q66PV0 and M4R4D0), *A. pulchra* (B5T1L1), *G. tenuidens* (Q95P04), *G. djiboutiensis* (A8CLL3), and *G. fascicularis* (A8CLV6), and FPs of *A. digitifera* (A0A1S7IWH7, A0A1S7IWI5, and A0A1S7IWI6). Although these CPs have a maximum absorbance at a wavelength of approximately 580 nm, they did not produce any fluorescence (Alieva et al., 2008). Furthermore, the amino acid sequences of plutCP and the other five proteins were compared with those of several known FPs, DsRed (Q2WG74), KAEDE (Q8I6J8), asFP595 (Q9GZ28), and other proteins listed in Table 1 by Alieva et al., 2008 (Figure 7). Surprisingly, all six proteins found in this study were identified as nonfluorescent CPs, unlike other RFPs, confirming that plutCP is a type of nonfluorescent CP that belongs to clade B in Alieva’s classification. Clade B mainly consists of purple and blue nonfluorescent CPs, and their genes have been found in Acroporidae, Pocilloporidae, Poritidae, Faviidae, Pectinidae, Oculinidae, and Dendrophyliidae. In this study, novel CP genes from *P. lutea* were added to this clade, and plutCP isolated from coral tissue. Finally, to confirm that the protein was indeed a nonfluorescent CP, we examined the corals using optical and fluorescence microscopy (Figure 8). A red-purple color was found throughout the coenosarc, and was particularly intense in color, whereas the tips of the tentacles were pale. In contrast, fluorescence observations revealed strong red fluorescence at the tips of the tentacles and almost no fluorescence in the coenosarc. Fluorescence images were obtained by filtering through a 600-nm bandpass filter attached under the objective lens of the microscope. Therefore, the red fluorescence of chlorophyll that is predominantly detected at a wavelength of 680 nm was removed; thus, the red fluorescence detected was most likely because of RFP, which emits fluorescence at wavelengths below 600 nm. As a reference, similar observations in healthy corals showed almost no fluorescence, suggesting that the detected fluorescence was a result of RFP and not chlorophyll. Thus, the protein responsible for the reddish-purple discoloration found in PPR was different from a RFP and confirmed to be an independent, nonfluorescent CP. These results indicate that CPs were distributed uniformly across the surface of the coral colonies mainly in the coenosarc, and the localization of the CPs was different from that of RFP. The molecular weight of plutCP extracted from massive *Porites* spp. manifesting PPRs was different in the SDS-PAGE (25 kDa), amino acid sequence (24,870 Da), and gel-filtration chromatography (49 kDa), and this result can be attributed to the formation of a dimer under native conditions, similar to that observed for GFP (Ward and Cormier, 1979). Ahmed et al. (2022) also reported that other CPs (gfasPurple, amilCP, spisPink, and eforRed) form dimers in solution, based on X-ray crystal structure analysis.

The present study demonstrated that CPs were encoded in the genome of *Porites* corals (*P. astreoides*, *P. australiensis*, and *P. lobata*), and the same CP expressed in different morphologies of PPRs (Ps and Pp), associated with the pink pigmentation response of inflamed tissues. Three presumed CP sequences were also identified in *A. digitifera* (Shinzato et al., 2012). These sequences were also included in the same clade as the six *Porites* sequences obtained in our study, which supports the present results as the 6 detected proteins beat similar properties as non-fluoresce and the formation of dimers. Moreover, in addition to plutCP synthesized by *Porites* spp. corals, a thioredoxin-like protein that is linked to redox signaling was expressed at high levels in the Ps lesions (Figure 5, Band 3).

The CP of *Porites* spp. was detected only in tissues that manifested PPRs. In relation to other types of CPs, Shinzato et al. (2012) reported that the three CPs of *A. digitifera* were highly expressed in the developmental stages but very poorly in adults. Studies by Takahashi-Kariyazono et al. (2018) also showed that FPs in middle-/long-wavelength emissions (519, 606, and 613 nm) and CPs were important for *Acropora* larvae, but were not expressed in adults. However, Smith et al. (2013) and Gittins et al. (2015) reported that high-level expression of CPs in adult corals (*Acropora nobilis* and *A. millepora*) was correlated with the reduction of photodamage under acute light stress, confirming the photoprotective function of CPs. This, together with the results of electrophoresis in the present study, show *Porites* spp. corals manifesting PPR lesions expressed high levels of CPs rather than in healthy tissues.

The PPRs in *Porites* spp. corals are highly distributed in the apical area of the colony, which is impacted by light in the shallow reefs of Okinawa during the low-tide period, in contrast to those medial and basal areas of the colony which are self-shaded (Yucharoen, 2016). The high production of CPs in the restricted PPR lesions reflects a focalized immune response of these corals to environmental stressors: biotic as the penetration of boring organisms, and more importantly, abiotic factors such as the salinity, the height of low tide, the time of exposure to air, and nutrient concentration, among others (In Yucharoen, 2016, pg.41, Table 5). In addition, RFPs and CPs coexist in the PPR lesions, but their distribution differs within the coral tissue, mainly coenosarc for CPs and tip of polyps’ tentacles for RFPs. CPs and their homologs have been suggested to serve as coral health indicators, as they usually protect cells against free radicals, and CPs likely also play important roles in the coral immune system and photoprotection, similar to that of RFPs. Palmer et al. (2009a) demonstrated that various CPs may act as antioxidants in coral hosts, showing a greater ability to scavenge hydrogen peroxide in inflamed tissues than healthy tissues. Correspondingly, the current study found high hydrogen peroxide-scavenging rates in *Porites* spp. corals tissues affected by PPR confirming this hypothesis. Several cases, such as dark-blue pigment in *A. hemprichii* (Rinkevich et al., 1994), pink-blue spot syndrome in *A. eurystoma* (Bongiorni and Rinkevich, 2005), dark spot syndromes in *Porites*, *Siderastrea*, and *Montastraea* (Cervino et al., 2001; Borger, 2005), and purple pigment in octocorals (Mullen et al., 2006), have been reported, but information about the presence and roles of CPs in *Porites* corals is scarce. D’Angelo et al. (2012) proposed that coloration changes in hosts expressing GFP-like proteins in response to wounding and infestation. Yucharoen (2016) reported a remarkable increase in CPs in relation to high HSA. Taken together, these studies indicate that massive *Porites* may produce CPs as an immune response against oxidative stress promoted by several environmental disturbances (biotic and abiotic), resulting in PPR lesions. Therefore, it can be concluded that the CP isolated from *Porites*. in the present study might possess antioxidant activity, contributing to the coral defense mechanism against ROS induced by various stresses (Yucharoen, 2016), and opportunistic infection in coral tissues displaying PPRs.

Although *Porites* corals are tolerant species that can adapt to various environments (Cantin and Lough, 2014), they occasionally show sensitivity to environmental stresses (Raymundo et al., 2005). Our results indicate that PPRs on *Porites* spp. in the area of Sesoko Lagoon could be promoted by environmental stressors and/or mechanical injuries caused by fish bites or boring organisms. CP, which is specifically expressed in PPR lesions, may serve as an antioxidant in the affected coral tissue. Furthermore, CP and RFP showed differential distribution within the PPR lesion, suggesting that CP, which is uniformly distributed in the coenosarc and covers a wide surface area, may have a more important role as an antioxidant than that of RFP, which is present only at the tips of tentacles.

This is the first study to report the role and distribution of CPs in corals manifesting PPR lesions. We believe that these findings will aid our understanding of PPR formation mechanism, which is an important subject in coral disease research. In addition, we describe, for the first time, the coexistence of nonfluorescent proteins and FPs in massive *Porites* spp. manifesting PPRs. Despite the PPR morphology (Ps or Pp) of *Porites* spp., the corals expressed the same type of CP. CPs have potential applications as genetic markers and cellular biosensors because of their ease of detection (Liljeruhm et al., 2018). The protocol used in this study, including genome analysis and microscopic observation using different filters, could be applied in future studies of pigments that commonly manifest in corals affected by other diseases. Therefore, this method opens the possibility to a deeper understanding of coral disease mechanisms in response to changing environment.

## 5 Compliance

Permission for coral sampling was obtained from Okinawa Prefecture (permit No. 30–55). The permit period was from 20 July 2018 to 19 July 2019.

## Data Availability

The original contributions presented in the study are included in the article/Supplementary Material, further inquiries can be directed to the corresponding author.

## References

[B1] Aeby G. S. (1992). The potential effect the ability of a coral intermediate host to regenerate has had on the evolution of its association with a marine parasite. Proceeding of the Seventh International Coral Reef Symposium, Guam, 1992, 2.

[B2] Aeby G. S. (2003). Corals in the genus *Porites* are susceptible to infection by a larval trematode. Coral Reefs 22, 216. 10.1007/s00338-003-0310-9

[B3] Aeby G. S. Williams G. J. Franklin E. C. Kenyon J. Cox E. F. Coles S. (2011). Patterns of coral disease across the Hawaiian Archipelago: relating disease to environment. PLoS ONE 6, e20370. 10.1371/journal.pone.0020370 21655248 PMC3105043

[B4] Ahmed F. H. Caputo A. T. French N. G. Peat T. S. Whitfield J. Warden A. C. (2022). Over the rainbow: structural characterization of the chromoproteins gfasPurple, amilCP, spisPink and eforRed. Acta Crystallogr. D. Struct. Biol. 78 (Pt 5), 599–612. 10.1107/S2059798322002625 35503208 PMC9063845

[B5] Alieva N. O. Konzen K. A. Field S. F. Meleshkevitch E. A. Hunt M. E. Beltran-Ramirez V. (2008). Diversity and evolution of coral fluorescent proteins. PLoS ONE 3, e2680. 10.1371/journal.pone.0002680 18648549 PMC2481297

[B6] Arya R. Mallik M. Lakhotia S. C. (2007). Heat shock genes - integrating cell survival and death. J. Biosci. 32, 595–610. 10.1007/s12038-007-0059-3 17536179

[B7] Beeden R. Willis B. Raymundo L. Page C. Weil E. (2008). Underwater cards for assessing coral health on indo-pacific reefs - Resources - CCRES.

[B8] Benzoni F. Galli P. Pichon M. (2010). Pink spots on *Porites*: not always a coral disease. Coral Reefs 29, 153. 10.1007/s00338-009-0571-z

[B9] Bollati E. Lyndby N. H. D'Angelo C. Kühl M. Wiedenmann J. Wangpraseurt D. (2022). Green fluorescent protein-like pigments optimise the internal light environment in symbiotic reef-building corals. eLife 11, e73521. 10.7554/eLife.73521 35801683 PMC9342951

[B10] Bongiorni L. Rinkevich B. (2005). The pink-blue spot syndrome in *Acropora eurystoma* (Eilat, Red Sea): a possible marker of stress? Zoology 108, 247–256. 10.1016/j.zool.2005.05.002 16351972

[B11] Borger J. L. (2005). Dark spot syndrome: a scleractinian coral disease or a general stress response? Coral Reefs 24, 139–144. 10.1007/s00338-004-0434-6

[B12] Bou-Abdallah F. Chasteen N. D. Lesser M. P. (2006). Quenching of superoxide radicals by green fluorescent protein. Biochimica Biophysica Acta - General Subj. 1760, 1690–1695. 10.1016/j.bbagen.2006.08.014 PMC1764454 17023114

[B13] Bradford M. M. (1976). A rapid and sensitive method for the quantitation of microgram quantities of protein utilizing the principle of protein-dye binding. Anal. Biochem. 72, 248–254. 10.1006/abio.1976.9999 942051

[B14] Bridges M. C. Woodley C. M. Peters E. C. May L. A. Galloway S. B. (2020). Expression and characterization of a bright far-red fluorescent protein from the pink-pigmented tissues of *Porites lobata* . Mar. Biotechnol. 22, 67–80. 10.1007/s10126-019-09931-9 31853751

[B15] Cantin N. E. Lough J. M. (2014). Surviving coral bleaching events: *Porites* growth anomalies on the Great Barrier Reef. PLoS One 9, e88720. 10.1371/journal.pone.0088720 24586377 PMC3929565

[B16] Cervino J. Goreau T. J. Nagelkerken I. Smith G. W. Hayes R. (2001). Yellow band and dark spot syndromes in Caribbean corals: distribution, rate of spread, cytology, and effects on abundance and division rate of zooxanthellae. Hydrobiologia 460, 53–63. 10.1023/A:1013166617140

[B17] Cottrell J. S. (2011). Protein identification using MS/MS data. J. Proteomics 74, 1842–1851. 10.1016/j.jprot.2011.05.014 21635977

[B18] D’Angelo C. Smith E. G. Oswald F. Burt J. Tchernov D. Wiedenmann J. (2012). Locally accelerated growth is part of the innate immune response and repair mechanisms in reef-building corals as detected by green fluorescent protein (GFP)-like pigments. Coral Reefs 31, 1045–1056. 10.1007/s00338-012-0926-8

[B19] Dereeper A. Audic S. Claverie J. M. Blanc G. (2010). BLAST-EXPLORER helps you building datasets for phylogenetic analysis. BMC Evol. Biol. 10, 8. 10.1186/1471-2148-10-8 20067610 PMC2821324

[B20] Dereeper A. Guignon V. Blanc G. Audic S. Buffet S. Chevenet F. (2008). Phylogeny.fr: robust phylogenetic analysis for the non-specialist. Nucleic acids Res. 36, W465–W469. 10.1093/nar/gkn180 18424797 PMC2447785

[B21] Gittins J. R. D’Angelo C. Oswald F. Edwards R. J. Wiedenmann J. (2015). Fluorescent protein-mediated colour polymorphism in reef corals: multicopy genes extend the adaptation/acclimatization potential to variable light environments. Mol. Ecol. 24, 453–465. 10.1111/mec.13041 25496144 PMC4949654

[B22] Han Y. Ma B. Zhang K. (2004).SPIDER: software for protein identification from sequence tags with *de novo* sequencing error. Proceedings - 2004 IEEE Computational Systems Bioinformatics Conference, CSB 2004. IEEE Computer Society, 206–215. 10.1109/csb.2004.1332434 16448014

[B23] Hughes T. P. Kerry J. T. Baird A. H. Connolly S. R. Dietzel A. Eakin C. M. (2018). Global warming transforms coral reef assemblages. Nature 556 (7702), 492–496. 10.1038/s41586-018-0041-2 29670282

[B24] Jin Y. K. Lundgren P. Lutz A. Raina J. B. Howells E. J. Paley A. S. (2016). Genetic markers for antioxidant capacity in a reef-building coral. Sci. Adv. 2 (5), e1500842. 10.1126/sciadv.1500842 27386515 PMC4928996

[B25] Johannes R. E. Wiebe W. J. (1970). Method for determination of coral tissue biomass and composition. Limnol. Oceanogr. 15, 822–824. 10.4319/lo.1970.15.5.0822

[B26] Johnson J. V. Exton D. A. Dick J. T. A. Oakley J. Jompa J. Pincheira-Donoso D. (2022). The relative influence of sea surface temperature anomalies on the benthic composition of an Indo-Pacific and Caribbean coral reef over the last decade. Ecol. Evol. 12 (9), e9263. 10.1002/ece3.9263 PMC9448965 36091340

[B27] Kashimoto R. Hisata K. Shinzato C. Satoh N. Shoguchi E. (2021). Expansion and diversification of fluorescent protein genes in fifteen *Acropora* species during the evolution of acroporid corals. Genes. 12 (3), 397. 10.3390/genes12030397 33799612 PMC8001845

[B28] Kenkel C. D. (2008). Coral disease: baseline surveys in the andaman sea and gulf of Thailand. Phuket Mar. Biol. Cent. Res. Bull. 53, 43–53.

[B29] Krause G. H. Weis E. (1991). Chlorophyll fluorescence and photosynthesis: the basics. Annu. Rev. Physiol. Plant Mol. Biol. 42, 313–349. 10.1146/annurev.pp.42060191.001525

[B30] Kritsanapuntu S. Angkhananukroh P. (2014). Coral disease prevalence in samui Island and the adjacent islands, southern part of the gulf of Thailand | international network for natural Sciences INNSPUB - academia.edu. J. Biodivers. Environ. Sci. 5, 158–165.

[B31] Kubomura T. Wee H. B. Reimer J. D. (2021). Investigating incidence and possible causes of pink and purple pigmentation response in hard coral genus *Porites* around Okinawajima Island, Japan. Regional Stud. Mar. Sci. 41, 101569. 10.1016/j.rsma.2020.101569

[B32] Kumar J. S. Y. Geetha S. Satyanarayana C. Venkataraman K. Kamboj R. D. (2014). Observations on coral diseases in marine national park, gulf of kachchh. Scholars Acad. J. Biosci. 2, 370–373. 10.36347/sajb.2014.v02i06.002

[B33] Labas Y. A. Gurskaya N. G. Yanushevich Y. G. Fradkov A. F. Lukyanov K. A. Lukyanov S. A. (2002). Diversity and evolution of the green fluorescent protein family. Proc. Natl. Acad. Sci. U. S. A. 99, 4256–4261. 10.1073/pnas.062552299 11929996 PMC123635

[B34] Laemmli U. K. (1970). Cleavage of structural proteins during the assembly of the head of bacteriophage T4. Nature 227, 680–685. 10.1038/227680a0 5432063

[B35] Lanneau D. Brunet M. Frisan E. Solary E. Fontenay M. Garrido C. (2008). Heat shock proteins: essential proteins for apoptosis regulation. J. Cell. Mol. Med. 12, 743–761. 10.1111/j.1582-4934.2008.00273.x 18266962 PMC4401125

[B36] Liew Y. J. Aranda M. Voolstra C. R. (2016). Reefgenomics.Org - a repository for marine genomics data. Database J. Biol. databases curation 2016, baw152. 10.1093/database/baw152 PMC5199144 28025343

[B37] Liljeruhm J. Funk S. K. Tietscher S. Edlund A. D. Jamal S. Wistrand-Yuen P. (2018). Engineering a palette of eukaryotic chromoproteins for bacterial synthetic biology. J. Biol. Eng. 12, 8. 10.1186/s13036-018-0100-0 29760772 PMC5946454

[B38] Lukyanov K. A. Fradkov A. F. Gurskaya N. G. Matz M. V. Labas Y. A. Savitsky A. P. (2000). Natural animal coloration can be determined by a nonfluorescent green fluorescent protein homolog. J. Biol. Chem. 275, 25879–25882. 10.1074/jbc.C000338200 10852900

[B39] Ma B. Zhang K. Hendrie C. Liang C. Li M. Doherty-Kirby A. (2003). PEAKS: powerful software for peptide *de novo* sequencing by tandem mass spectrometry. Rapid Commun. Mass Spectrom. 17, 2337–2342. 10.1002/rcm.1196 14558135

[B40] Matz M. V. Fradkov A. F. Labas Y. A. Savitsky A. P. Zaraisky A. G. Markelov M. L. (1999). Fluorescent proteins from nonbioluminescent Anthozoa species. Nat. Biotechnol. 17, 969–973. 10.1038/13657 10504696

[B41] Miyawaki A. (2002). Green fluorescent protein-like proteins in reef anthozoa animals. Cell. Struct. Funct. 27, 343–347. 10.1247/csf.27.343 12502888

[B42] Mohamed A. R. Ali A.-H. A. M. Abdel-Salam H. A. (2012). “Status of coral reef health in the northern Red Sea, Egypt,” in 12th international coral reef symposium, 5.

[B43] Montalbetti E. Biscéré T. Ferrier-Pagès C. Houlbrèque F. Orlandi I. Forcella M. (2021). Manganese benefits heat-stressed corals at the cellular level. Front. Mar. Sci. 29 June 2021 Sec. Aquat. Physiol. 8–2021. 10.3389/fmars.2021.681119

[B44] Mullen K. M. Harvell C. D. Alker A. P. Dube D. Jordán-Dahlgren E. Ward J. R. (2006). Host range and resistance to aspergillosis in three sea fan species from the Yucatan. Mar. Biol. 149, 1355–1364. 10.1007/s00227-006-0275-7

[B45] Nishihira M. (2004). “Hermatypic corals of Japan,” in: Coral Reefs of Japan, eds. Ministry of the Environment and The Japanese Coral Reef Society (Tokyo, Japan: Ministry of the Environment), 10–13.

[B46] Palmer C. V. Modi C. K. Mydlarz L. D. (2009a). Coral fluorescent proteins as antioxidants. PLoS ONE 4, e7298. 10.1371/journal.pone.0007298 19806218 PMC2752795

[B47] Palmer C. V. Roth M. S. Gates R. D. (2009b). Red fluorescent protein responsible for pigmentation in trematode-infected *Pontes compressa* tissues. Biol. Bull. 216, 68–74. 10.1086/BBLv216n1p68 19218493

[B48] Putchim L. Yamarunpattana C. Phongsuwan N. (2012). Observations of coral disease in *Porites lutea* in the Andaman Sea following the 2010 bleaching. Phuket Mar. Biol. Cent. Res. Bull. 71, 57–62.

[B49] Ravindran J. Raghukumar C. (2002). Pink line syndrome (PLS) in the scleractinian coral *Porites lutea* . Coral Reefs 21, 252. 10.1007/s00338-002-0247-4

[B50] Ravindran J. Raghukumar C. (2006). Histological observations on the scleractinian coral *Porites lutea* affected by pink-line syndrome. Curr. Sci. 90, 720–724. 10.2307/24089122

[B51] Raymundo L. J. Resell K. B. Reboton C. T. Kaczmarsky L. (2005). Coral diseases on Philippine reefs: genus *Porites* is a dominant host. Dis. Aquatic Org. 64, 181–191. 10.3354/dao064181 15997816

[B52] Rinkevich B. Frank U. Bak R. P. M. Müller W. E. G. (1994). Alloimmune responses between *Acropora hemprichi* conspecifics: nontransitive patterns of overgrowth and delayed cytotoxicity. Mar. Biol. 118, 731–737. 10.1007/BF00347522

[B53] Satoh N. Kinjo K. Shintaku K. Kezuka D. Ishimori H. Yokokura A. (2021). Color morphs of the coral, *Acropora tenuis*, show different responses to environmental stress and different expression profiles of fluorescent-protein genes. G3 11 (2), jkab018. 10.1093/g3journal/jkab018 33621334 PMC8022974

[B54] Séré M. G. Chabanet P. Turquet J. Quod J. P. Schleyer M. H. (2015). Identification and prevalence of coral diseases on three Western Indian Ocean coral reefs. Dis. Aquatic Org. 114, 249–261. 10.3354/dao02865 26036832

[B55] Seveso D. Arrigoni R. Montano S. Maggioni D. Orlandi I. Berumen M. L. (2020). Investigating the heat shock protein response involved in coral bleaching across scleractinian species in the central Red Sea. Coral Reefs 39, 85–98. 10.1007/s00338-019-01878-6

[B56] Seveso D. Montano S. Maggioni D. Pedretti F. Orlandi I. Galli P. (2018). Diel modulation of Hsp70 and Hsp60 in corals living in a shallowreef. Coral Reefs 37, 801–806. 10.1007/s00338-018-1703-0

[B57] Shinzato C. Shoguchi E. Tanaka M. Satoh N. (2012). Fluorescent protein candidate genes in the coral *Acropora digitifera* genome. Zoological Sci. 29, 260–264. 10.2108/zsj.29.260 22468836

[B58] Smith E. G. D’Angelo C. Salih A. Wiedenmann J. (2013). Screening by coral green fluorescent protein (GFP)-like chromoproteins supports a role in photoprotection of zooxanthellae. Coral Reefs 32, 463–474. 10.1007/s00338-012-0994-9

[B59] Sully S. Burkepile D. E. Donovan M. K. Hodgson G. van Woesik R. (2019). A global analysis of coral bleaching over the past two decades. Nat. Commun. 10 (1), 1264–1265. 10.1038/s41467-019-09238-2 30894534 PMC6427037

[B60] Takahashi-Kariyazono S. Sakai K. Terai Y. (2018). Presence–absence polymorphisms of highly expressed FP sequences contribute to fluorescent polymorphisms in *Acropora digitifera* . Genome Biol. Evol. 10, 1715–1729. 10.1093/gbe/evy122 30016429 PMC6048989

[B61] Thinesh T. Mathews G. Edward J. K. P. (2011). Coral disease prevalence in the Palk Bay, Southeastern India – with special emphasis to black band. Indian J. Geo-Marine Sci. 40, 813–820.

[B62] Thummasan M. Casareto B. E. Ramphul C. Suzuki T. Toyoda K. Suzuki Y. (2021). Physiological responses (Hsps 60 and 32, caspase 3, H_2_O_2_ scavenging, and photosynthetic activity) of the coral *Pocillopora damicornis* under thermal and high nitrate stresses. Mar. Pollut. Bull. 171, 112737. 10.1016/j.marpolbul.2021.112737 34298325

[B63] Verkhusha V. V. Lukyanov K. A. (2004). The molecular properties and applications of Anthozoa fluorescent proteins and chromoproteins. Nat. Biotechnol. 22, 289–296. 10.1038/nbt943 14990950

[B64] Veron J. E. N. (1992). Conservation of biodiversity: a critical time for the hermatypic corals of Japan. Coral Reefs 11, 13–21. 10.1007/BF00291930

[B65] Ward W. W. Cormier M. J. (1979). An energy transfer protein in coelenterate bioluminescence. Characterization of the Renilla green-fluorescent protein. J. Biol. Chem. 254 (3), 781–788. 10.1016/S0021-9258(17)37873-0 33175

[B66] Weil E. Irikawa A. Casareto B. Suzuki Y. (2012). Extended geographic distribution of several Indo-Pacific coral reef diseases. Dis. Aquatic Org. 98, 163–170. 10.3354/dao02433 22436464

[B67] Weil E. Rogers C. S. Croquer A. (2016). “Octocoral diseases in a changing ocean,” in Marine animal forests. Editors Rossi S. Bramanti L. Gori A. Orejas Saco del Valle C. (Springer International Publishing), 1–55.

[B68] Wiedenmann J. Elke C. Spindler K. D. Funke W. (2000). Cracks in the β-can: fluorescent proteins from *Anemonia sulcata* (anthozoa, actinaria). Proc. Natl. Acad. Sci. U. S. A. 97, 14091–14096. 10.1073/pnas.97.26.14091 11121018 PMC18876

[B69] Willis B. L. Page C. A. Dinsdale E. A. (2004). Coral disease on the great barrier reef. Coral health and disease. Berlin Heidelberg: Springer, 69–104.

[B70] Yucharoen M. (2016). Pink pigmentation response during recovery period after coral bleaching. dissertation/PhD Thesis. Shizuoka (Japan): Shizuoka University. 10.14945/00009914

