# Supplementary material for: Coexistence of nonfluorescent chromoproteins and fluorescent proteins in massive Porites spp. corals manifesting a pink pigmentation response
Source: Front Physiol. 2024 Jun 17;15:1339907. doi: 10.3389/fphys.2024.1339907 (PMC11215327; doi:10.3389/fphys.2024.1339907)
Supplement: Supplementary file 1 [file Table1.pdf]

Supplementary Table S1: Maximum quantum yield of Symbiodiniaceae of the coral colonies exhibiting PPR as pink patch (Pp), and the healthy colony (H).

	F0	Fv/Fm
Pp	345	0.400
	335	0.388
	343	0.413
	404	0.422
	354	0.437
	303	0.484
	372	0.505
	440	0.429
	361	0.421
	438	0.440
H	583	0.506
	597	0.528
	589	0.534
	618	0.499
	607	0.523
	623	0.510
	612	0.540
	616	0.523
	567	0.516
	590	0.532
