# Supplementary material for: Coexistence of nonfluorescent chromoproteins and fluorescent proteins in massive Porites spp. corals manifesting a pink pigmentation response
Source: Front Physiol. 2024 Jun 17;15:1339907. doi: 10.3389/fphys.2024.1339907 (PMC11215327; doi:10.3389/fphys.2024.1339907)
Supplement: Supplementary file 2 [file Table2.pdf]

Supplementary Table S2: Antixidant enzymatic activity of the coral colonies exhibiting PPR as pink patch (Pp), and the healthy colony (H).

| | A240 (t=0) | A240 (t=30) | Protein (mg/ml) | Activity (nmol HPO/mg protein/min) |
|---|---|---|---|---|
| Pp | 0.315 | 0.299 | 1.289 | 1.924 |
| | 0.302 | 0.285 | 1.355 | 1.945 |
| | 0.320 | 0.304 | 1.308 | 1.896 |
| | 0.335 | 0.321 | 1.152 | 1.884 |
| | 0.322 | 0.307 | 1.267 | 1.836 |
| | 0.318 | 0.299 | 1.615 | 1.824 |
| | 0.322 | 0.308 | 1.152 | 1.884 |
| | 0.320 | 0.305 | 1.296 | 1.794 |
| | 0.318 | 0.302 | 1.314 | 1.888 |
| H | 0.536 | 0.526 | 3.380 | 0.459 |
| | 0.526 | 0.516 | 3.031 | 0.512 |
| | 0.518 | 0.508 | 2.955 | 0.525 |
| | 0.465 | 0.457 | 2.992 | 0.415 |
| | 0.461 | 0.452 | 3.165 | 0.441 |
| | 0.484 | 0.477 | 3.012 | 0.360 |
| | 0.655 | 0.647 | 2.928 | 0.424 |
| | 0.646 | 0.639 | 2.868 | 0.378 |
| | 0.672 | 0.663 | 2.860 | 0.488 |
